# Discovery of novel molecular characteristics and cellular biological properties in ameloblastoma

**DOI:** 10.1002/cam4.2931

**Published:** 2020-02-25

**Authors:** Sayuri Kondo, Akinobu Ota, Takayuki Ono, Sivasundaram Karnan, Md Wahiduzzaman, Toshinori Hyodo, Md Lutfur Rahman, Kunihiro Ito, Akifumi Furuhashi, Tomio Hayashi, Hiroyuki Konishi, Shinobu Tsuzuki, Yoshitaka Hosokawa, Yoshiaki Kazaoka

**Affiliations:** ^1^ Department of Oral and Maxillofacial Surgery Aichi Medical University Hospital Nagakute Japan; ^2^ Department of Biochemistry Aichi Medical University School of Medicine Nagakute Japan

**Keywords:** ameloblastoma, gene expression, IGF2, proliferation, signaling pathway, TLR2

## Abstract

Ameloblastoma is a rare odontogenic benign tumor accounting for less than 1% of head and neck tumors. Advanced next generation sequencing (NGS) analyses identified high frequency of *BRAF* V600E and *SMO* L412F mutations in ameloblastoma. Despite the existence of whole genomic sequence information from patients with ameloblastoma, entire molecular signature of and the characteristics of ameloblastoma cells are still obscure. In this study, we sought to uncover the molecular basis of ameloblastoma and to determine the cellular phenotype of ameloblastoma cells with *BRAF* mutations. Our comparative cDNA microarray analysis and gene set enrichment analysis (GSEA) showed that ameloblastoma exhibited a distinct gene expression pattern from the normal tissues: KRAS‐responsive gene set is significantly activated in ameloblastoma. Importantly, *insulin like growth factor 2 (IGF2)*, a member of KRAS‐responsive genes, enhances the proliferation of an ameloblastoma cell line AMU‐AM1 with *BRAF* mutation. In addition, Toll‐like receptor 2 (TLR2) knockdown readily inactivated KRAS‐responsive gene sets as well as increases caspase activities, suggesting that TLR2 signaling may mediate cell survival signaling in ameloblastoma cells. Collectively, the findings may help to further clarify the pathophysiology of ameloblastoma and lead to the development of precision medicine for patients with ameloblastoma.

## Graphical abstract

The present study is the first to demonstrate novel molecular characteristics in ameloblastoma, where oncogenic gene signatures including KRAS‐responsive genes and EGFR‐related genes were significantly activated. Our findings may help to further clarify the pathophysiology of ameloblastoma and lead to the development of precision medicine for patients with ameloblastoma.

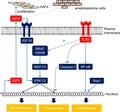

## 1 INTRODUCTION

Ameloblastoma is a rare odontogenic benign tumor, which accounts for less than 1% of head and neck tumors.^1^, ^2^, ^3^, ^4^, ^5^ To date, patients with ameloblastoma have been mainly managed by surgical operation. Although most of the patients with ameloblastoma can be cured after surgical resection of primary tumor, recurrence, and/or malignant transformation into ameloblastoma carcinoma have been observed in the patients with a range of risk.^5^, ^6^, ^7^, ^8^, ^9^, ^10^, ^11^, ^12^, ^13^, ^14^, ^15^ Effiom et al reported that 70% of ameloblastomas undergo malignant transformation and that metastasizing ameloblastomas account for 2% of benign ameloblastomas.^6^, ^7^ Rizzitelli et al reported that the incidence of malignant ameloblastoma was higher in men than in women and that it was higher in black people than in white people, using the Surveillance, Epidemiology, and End‐Results (SEER) database.^8^ Other long‐term follow‐up studies have also shown varying recurrence rates (4.5%‐29.3%) among ethnic groups.^5^, ^9^, ^10^, ^11^, ^12^, ^13^, ^14^, ^15^ Several studies have indicated that the initial conservative surgical management is closely associated with high recurrence of tumor.^15^ Radical surgical operation is currently recommended for curative therapy of ameloblastoma, while marginal resection, enucleation and curettage are sometimes desired for conservation and/or reconstruction of operative regions. The association between recurrence rate of ameloblastoma and the pathological classification remains unelucidated.

Advanced next generation sequencing (NGS) analyses identified the high frequency of *BRAF* V600E and *SMO* L412F mutations in ameloblastoma.^16^, ^17^, ^18^ In 2018, Gültekin et al reported novel somatic mutation of oncogenes and tumor suppressor genes in addition to BRAF and SMO mutations.^18^ Using cell culture model with established ameloblastoma cell lines, Nakao et al reported that fibroblast growth factor (FGF) −7 and −10 stimulates ameloblastoma proliferation.^19^ Sathi et al reported that secreted frizzled related protein‐2 is involved in the proliferation of ameloblastoma cells.^20^ These reports implicate the mitogen‐activated protein kinase pathway and/or sonic hedgehog signaling pathway in the pathogenesis of ameloblastoma cells.^21^ Heikinheimo et al reported a targeted microarray study focusing on the mRNA expression of 588 cancer‐related genes.^22^ Although a certain subset of genomic sequence information or specific gene expression regarding ameloblastoma patients have been explored, the comprehensive gene expression profiles related to ameloblastoma remains largely unknown. Additionally, the relationship between gene expression changes and genomic alterations in ameloblastoma is still obscure.

In this study, we performed comprehensive cDNA microarray analyses to investigate the characterization of the transcriptomic abnormalities in ameloblastoma using the patients‐derived ameloblastoma tumors and normal oral tissues. In addition, we established a novel ameloblastoma cell line AMU‐AM1 with *BRAF* V600E mutation. Furthermore, we examined the proliferative activities of secreted factors predominantly expressed in the ameloblastoma. The association of Toll‐like receptor 2 (TLR2) expression with the behavior of ameloblastoma cells is also discussed.

## 2 MATERIALS AND METHODS

### 2.1 Patients and tumor samples

This study was reviewed and approved by the relevant review boards of Aichi Medical University School of Medicine, Japan (No. 2016‐H103). Prior to initiating the study, informed consent was obtained from the parents or guardians of all the patients to use their samples for banking and subsequent molecular analyses. The study group consisted of nine patients with primary ameloblastoma, and the diagnoses were done by pathologists at Aichi Medical University Hospital, according to the histological classification of odontogenic tumors by the World Health Organization (WHO).^23^ In this study, fresh cold tissue samples of ameloblastoma tumors and/or normal gingiva obtained from nine patients were examined using cDNA microarray, real‐time RT‐PCR, and western blotting analysis. One tumor and one normal sample (0.5‐2 cm in diameter) were obtained from each patient using a scalpel. The patient characteristics are summarized in Table 1. All the ameloblastoma were located in the mandible, which is known as the most common tumor location of ameloblastoma worldwide.^10^, ^11^

**Table 1:** Summary of ameloblastoma patients analyzed in this study

Patient No.	Sex	Age	Type	Relapse	*BRAF*		*SMO*
					Exon 11	Exon 15	
1	M	44	Mandible	−	WT	V600E	WT
2	F	27	Mandible	−	WT	V600E	WT
3	M	12	Mandible	−	WT	V600E	WT
4	F	25	Mandible	−	WT	WT	WT
5	M	56	Mandible	−	WT	WT	WT
6	F	67	Mandible	−	WT	V600E	WT
7	F	19	Mandible	+	WT	V600E	WT
8	M	67	Mandible	−	WT	V600E	WT
9	F	44	Mandible	−	WT	V600E	WT

Note

Sanger sequencing analysis were performed using patients‐derived tumor DNA to examine gene mutation in *BRAF* exons 11 and 15 as well as *SMO*.

### 2.2 Establishment of an ameloblastoma cell line

The tumor tissues excised from ameloblastoma patients were minced and treated with collagenase IV (2 mg/mL), hyaluronidase (0.5 mg/mL) for 1 hour of shaking at 190 rpm, and further incubated with DNase (1 U/mL) for 15 minutes. After incubation, the cells were passed through a filter (100 μm) and were washed using RPMI1640 containing 10% of fetal bovine serum (FBS). The cells were maintained at 37°C in a 5% of CO_2_ atmosphere in 50:50 DMEM/F‐12 (Wako) medium containing 10% of FBS (Sigma‐Aldrich) and supplemented with penicillin‐streptomycin (100 μg/mL; Wako), hydrocortisone (100 μg/mL; Sigma‐Aldrich), insulin (2.5 μg/mL; Sigma‐Aldrich), EGF (10 μg/mL; Wako), and transferrin (2.5 μg/mL; Sigma‐Aldrich). To achieve immortalization, the cells were infected with human papilloma virus (E6‐7, Funakoshi) in the presence of polybrene (8 μg/mL) for 24 hours. After several passages, the growing cells were picked and the DNA sequences of the *BRAF* (Exon11 and Exon15) and *SMO* genes were examined using Sanger sequencing. The resulting established ameloblastoma cell line was designated AMU‐AM1 (from patient No.7). We simultaneously established immortalized patient‐derived fibroblast cell lines from patients No.5 (AM5‐F), No.7 (AM7‐F), and No.9 (AM9‐F), all of which have no *BRAF* gene mutation (Figure S1).

### 2.3 cDNA microarray analysis

Total RNA was isolated using TRIzol^TM^ reagent (Thermo Fisher Scientific KK) and NucleoSpin RNA (TaKaRa Bio Inc) with DNase treatment. The quantity and quality of total RNA were confirmed by using NanoDrop^TM^ 1000 (Thermo Fisher Scientific KK) with 260/230 and 260/280 ratios. To further verify the quality of RNA, we used RT‐PCR to examine DNA amplification using agarose gel electrophoresis. RT‐PCR analysis showed that gene amplification did not work well only in the case of Patient No. 9. Therefore, we did not perform cDNA microarray analysis for Patient No. 9. The experimental procedure for the cDNA microarray was based on the manufacturer's protocol (Agilent Technologies) and described previously.^24^, ^25^ In brief, cDNA synthesis and cRNA labeling with cyanine 3 (Cy3) dye were performed using the Agilent Low Input Quick Amp Labeling Kit (Agilent Technologies). The Cy3‐labeled cRNA was purified, fragmented, and hybridized on a Human Gene Expression 8 × 60 K v2 Microarray Chip containing 26 740 Entrez Gene RNAs using a Gene Expression Hybridization kit (Agilent Technologies). The raw and normalized microarray data have been submitted to the GEO database at NCBI (http://www.ncbi.nlm.nih.gov/geo/query/acc.cgi?acc=GSE132472; https://www.ncbi.nlm.nih.gov/geo/query/acc.cgi?acc=GSE132472). GSEA was performed according to the instructions.

### 2.4 Quantitative reverse transcription (qRT)‐PCR analysis

qRT‐PCR analysis was performed using SYBR Green I, as previously described.^26^ The primers used in this study are described in Table S1. Glyceraldehyde‐3‐phosphate dehydrogenase (*GAPDH*) was used as an internal control.

### 2.5 Western blot analysis

Western blot analysis was performed as described previously.^27^ The antibodies used in this study are described in Table S2. Immune complexes were detected using ImmunoStar LD (Wako Pure Chemical Industries, Ltd.) in conjunction with a LAS‐4000 image analyzer (GE Healthcare).

### 2.6 Cell viability (MTT) assay

The AMU‐AM1 cells were seeded in 96‐well culture plates (2 × 10^3^ cells/well). Next, the cells were incubated with medium containing the indicated concentrations of recombinant protein. After incubation for 72 hours, the 3‐(4,5‐dimethylthiazol‐2‐yl)‐2,5‐diphenyltetrazolium bromide (MTT) assay was performed as described previously.^28^ The absorbance at 595 nm was measured using a SpectraMax M5 spectrophotometer (Molecular Devices).

### 2.7 RNA interferences

The AMU‐AM1 cells were seeded in 6‐well culture plates (1 × 10^5^ cells/well). Next, the cells were transfected with 10 nmol/L of control siRNA, TLR2 siRNA‐1 (S168; 5′‐GCCUUGACCUGUCCAACAAtt), or TLR2 siRNA‐2 (S169; 5′‐GACUUAUCCUAUAAUUACUtt) using Lipofectamine 3000 reagents according to the manufacture's instruction (Thermo Fisher Scientific KK). After 48 hours of transfection, the cells were applied to the series of bioassays.

### 2.8 Statistical analysis

Results are expressed as mean ± SE. Statistical significance between groups was determined using one‐way ANOVA and student's *t* test. Statistical analyses were performed using SPSS 23.0 program (SPSS Inc).

## 3 RESULTS

### 3.1 Oncogenic signatures were significantly activated in ameloblastoma

**Figure 1:**
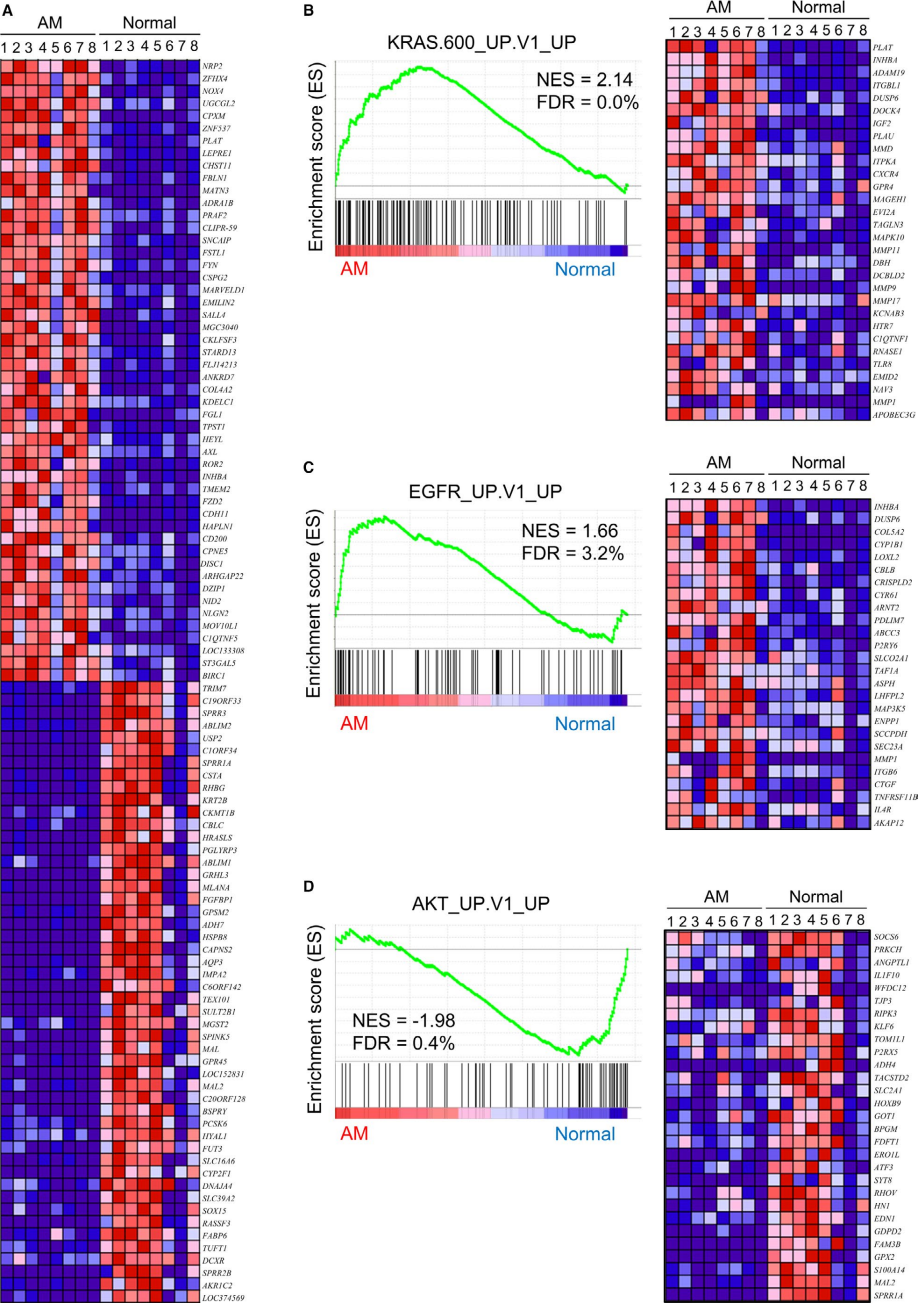
Activation of genes in the KRAS‐related and EGFR‐related gene sets in ameloblastoma tumor. cDNA microarray analysis was performed using total RNA, which was isolated from tumor lesions or normal oral tissues of eight individual patients with ameloblastoma. A, Heat map showing a distinct gene expression pattern in ameloblastoma tumors and corresponding normal oral tissues. B‐D, GSEA was conducted using GSEA v2.2.4 software and Molecular Signatures Database (Broad Institute). All raw data were formatted and applied to oncogenic signatures (C6). Representative enrichment plots of (B) KRAS.600_UP.V1_UP, (C) EGFR_UP.V1_UP, and (D) AKT_UP.V1_UP with corresponding heat map images of the indicated gene sets in the ameloblastoma tumors (AM) and/or normal tissues (Normal) are shown. Genes contributing to enrichment are shown in rows. Expression level is represented as a gradient from high (red) to low (blue). FDR, false discovery rate; NES, normalized enrichment score

To investigate the global gene expression changes in ameloblastoma, we performed cDNA microarray analyses on RNA isolated from patient‐derived ameloblastoma and from patient‐matched normal oral tissues. The patient information is summarized in Table 1. Heat map analysis revealed distinct gene expression patterns of ameloblastoma and normal oral tissues (Figure 1A). Fold change analysis showed that the expression of 204 genes was upregulated by >10.0 fold and the expression of 342 genes was downregulated by <0.1 fold in the ameloblastoma tumor compared to corresponding normal oral tissues (Tables S3 and S4). GSEAs of genes with oncogenic signatures (C6) showed a significant activation of KRAS‐responsive genes (Figure 1B) including, inhibin subunit beta A *(INHBA)*, *insulin like growth factor 2 (IGF2),* and matrix metalloproteinase 9 (*MMP9*), and EGFR‐induced genes (Figure 1C) including *INHBA*, collagen type V alpha 2 chain (*COL5A2*)*,* and connective tissue growth factor (*CTGF*, also known as cellular communication network factor 2, *CCN2*), along with significant inactivation of Akt signaling‐associated genes (Figure 1D), including small proline rich protein 1A (*SPRR1A*), S100 calcium binding protein A14 (*S100A14*), and activating transcriptional factor (*ATF3*) in ameloblastoma tumors compared to the normal tissues. In addition, GSEA showed significant activation or inactivation of tumorigenesis‐related gene sets (Tables S5 and S6). Interestingly, we found that TGF‐β signaling‐related genes including transforming growth factor beta 3 (*TGFB3*), snail family transcriptional repressor 1 (*SNAI1*), and inhibitor of DNA binding 3 (*ID3*) were significantly activated in ameloblastoma tumors (Figure S2).

### 3.2 Gene expression of KRAS‐responsive gene sets were significantly upregulated in ameloblastoma

**Figure 2:**
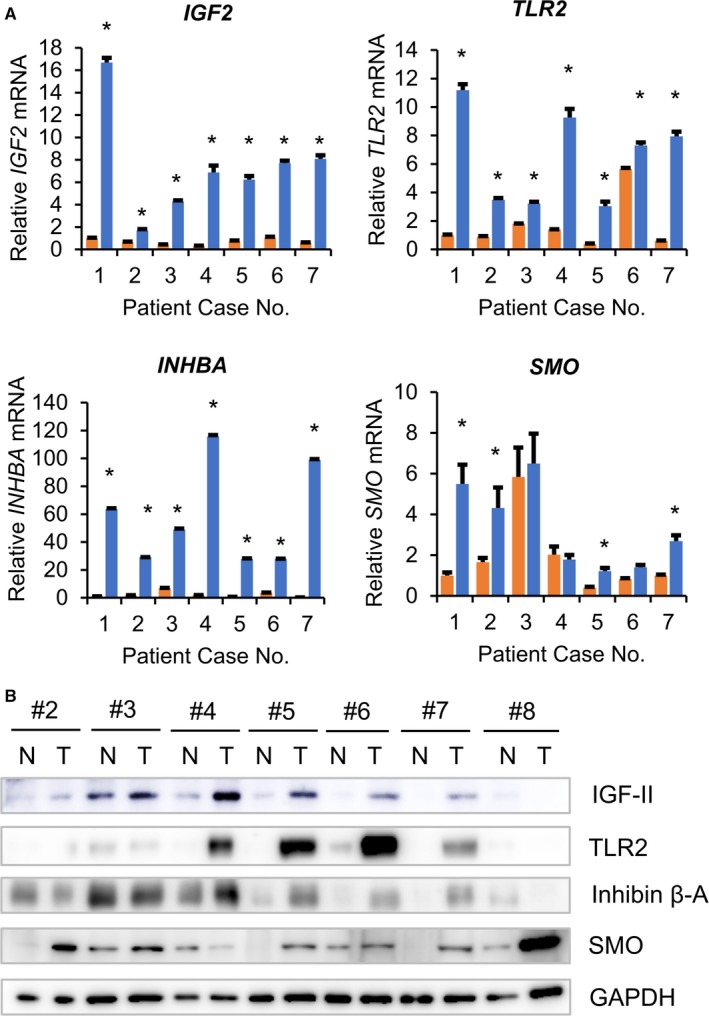
Upregulation of KRAS‐responsive genes IGF2, TLR2, and INHBA in ameloblastoma. A‐B, qRT‐PCR analysis (A) and western blot analysis (B) for the expression levels of *IGF2*, *TLR2*, *INHBA*, and *SMO* in ameloblastoma and normal oral tissues. A, The primers used for qRT‐PCR are shown in Table S1. Relative gene expression levels are shown after normalization to *GAPDH* mRNA expression. The data are expressed relative to the mRNA levels found in the corresponding normal tissue sample of Patient No.1, which were arbitrarily defined as 1. The values shown represent the mean ± SE (n = 3). B, Western blot analysis. The antibodies used for western blot analysis are shown in Table S2. GAPDH was used as an internal control. N, normal tissue; T, tumor. Asterisk (*) indicates statistically significant difference at *P* < .05 (n = 3)

To verify the microarray results, we examined the expression levels of KRAS‐responsive genes using qRT‐PCR analysis. This analysis showed that mRNA levels of *IGF2*, *TLR2*, and *INHBA* were significantly increased in all the ameloblastoma tumors compared to normal tissues, while mRNA levels of *SMO* partially increased in the ameloblastoma tumors of patients 1, 2, 5, and 7 (Figure 2A). Accordingly, western blot analysis showed that protein expression levels of IGF‐II, TLR2, and INHBA were significantly increased in the ameloblastoma tumors compared to normal tissues (Figure 2B). We also found that gene expression of amelotin (*AMTN*), which encodes a secreted protein specifically expressed during the late stages of enamel formation, greatly increased in five out of seven ameloblastoma tumors compared to normal tissues (Figure S3A). These results suggest that KRAS signaling is highly activated in ameloblastoma tumors compared to surrounding oral tissues.

### 3.3 Establishment and characterization of ameloblastoma cell line AMU‐AM1

**Figure 3:**
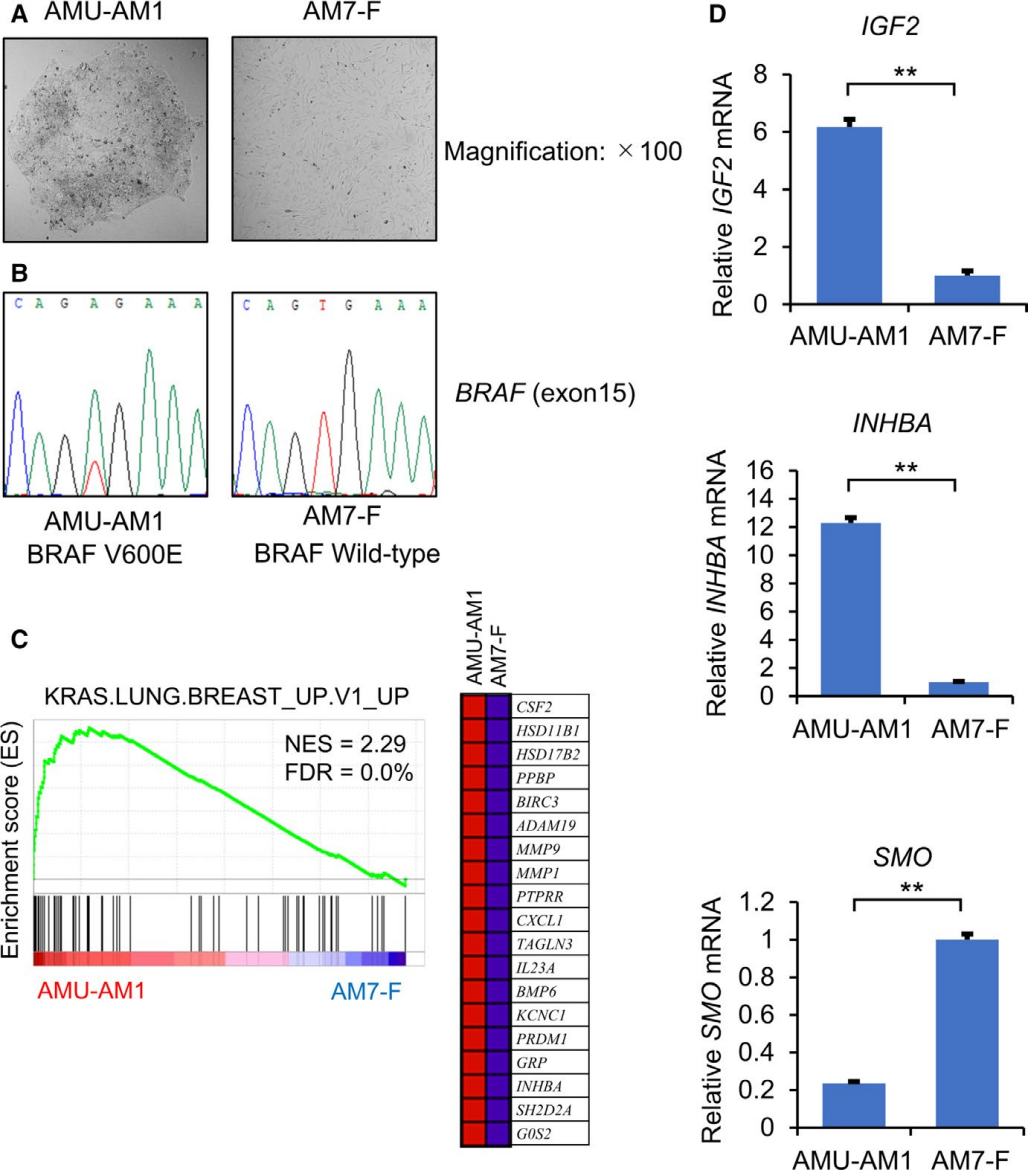
Gene expression profile in AMU‐AM1 cells. A, Cell morphology of AMU‐AM1 cells and the corresponding immortalized fibroblast AM7‐F cells under light microscope (magnification: ×100). B, Representative results of Sanger sequencing of the *BRAF* gene (Exon 15). C, cDNA microarray analysis was performed using total RNA, which was isolated from AMU‐AM1 and AM7‐F cells. GSEA was conducted as describing in Figure 1. KRAS.LUNG.BREAST_UP.V1_UP with corresponding heat map images of the indicated gene sets in the AMU‐AM1 and AM7‐F cells are shown. D, qRT‐PCR analysis of *IGF2*, *INHBA,* and *SMO* in AMU‐AM1 and AM7‐F cells. The data are expressed relative to the mRNA levels found in the AM7‐F cells, which were arbitrarily defined as 1. The values shown represent the mean ± SE (n = 3). Asterisk (**) indicates statistically significant difference at *P* < .005 (n = 3)

To further investigate the characterization of ameloblastoma cells, we established the ameloblastoma cell line AMU‐AM1 and the corresponding fibroblast cell line AM7‐F from ameloblastoma patient No.7 with *BRAF* V600E mutation (Figure 3A,B). We verified whether KRAS‐responsive genes are upregulated in the ameloblastoma cells and/or the ameloblastoma‐associated fibroblasts (AAF). Of note, GSEA of genes with oncogenic and HALLMARK gene signatures showed a significant activation of KRAS‐responsive genes (Figure 3C; eg *INHBA*, *MMP1*, and *IGF2*) in AMU‐AM1 cells, compared to AM7‐F cells. GSEA also showed a significant activation of β‐catenin (BCAT)‐related genes (Figure S4) including dickkopf WNT signaling pathway inhibitor 1 (*DKK1*), LIM domain only 2 (*LMO2*), and BCL2 interacting killer (*BIK*) in AMU‐AM1 cells. In addition, qRT‐PCR analysis showed that mRNA levels of *INHBA* as well as *IGF2* were significantly higher, whereas that of *SMO* is significantly lower in AMU‐AM1 cells compared with AM7‐F cells (Figure 3D). These results suggest that KRAS signaling pathway play an important role in AMU‐AM1 cells.

### 3.4 IGF2 is a candidate growth factor for ameloblastoma cells

**Figure 4:**
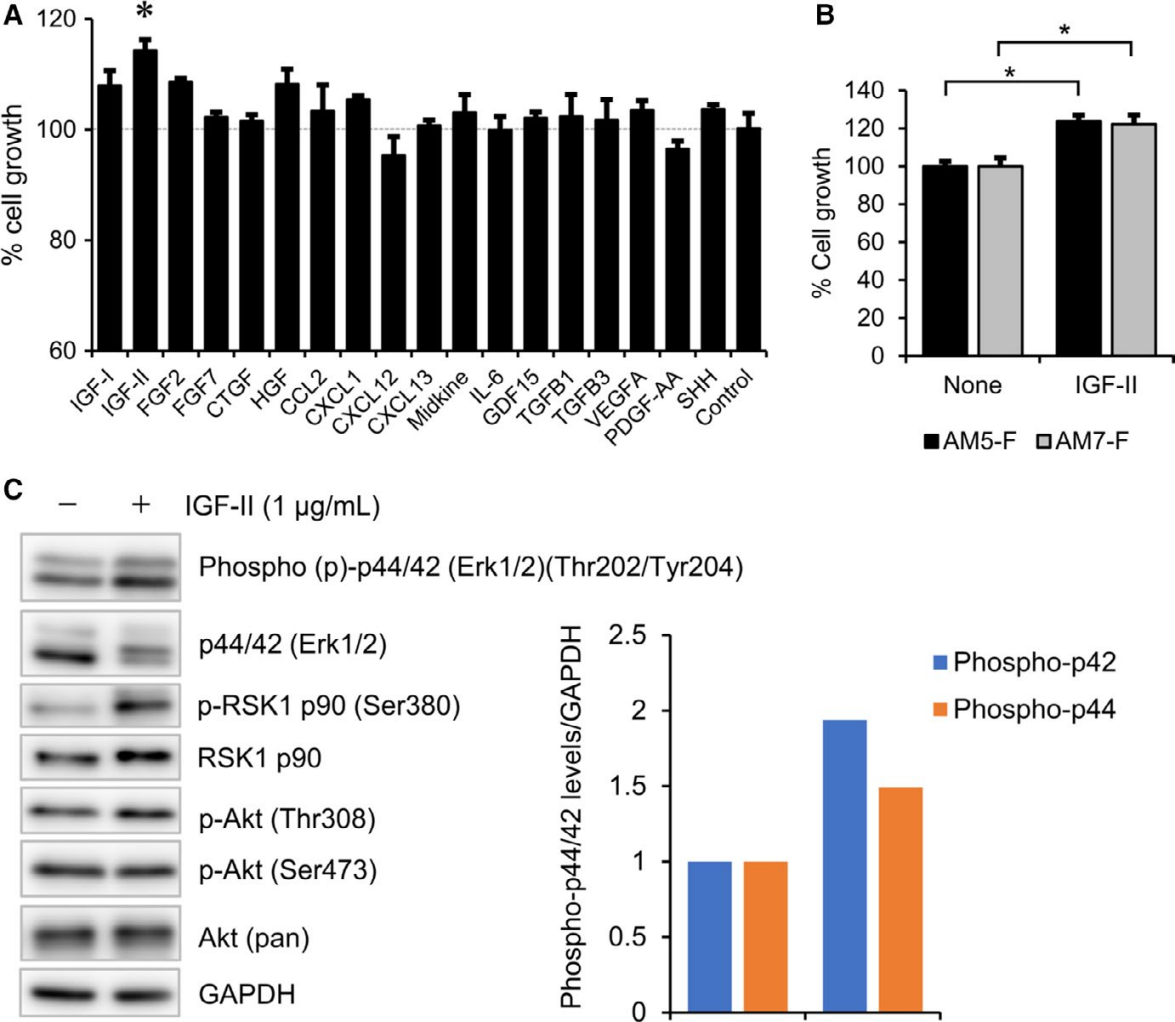
Effect of secreting factors on the proliferation of AMU‐AM1 cells. A, MTT assay showing the effect of various growth factors on the cell survival of AMU‐AM1 cells. AMU‐AM1 cells (2 × 10^3^ cells/well in a 96‐well plate) were treated with 100 ng/mL of the recombinant proteins for 72 h. Percentage growth rate of the cell clones was measured by performing the MTT assay. Data are expressed relative to the mean optic density (595 nm) of untreated cells, which was arbitrarily defined as 100%. Data are expressed as mean ± SE (n = 3). B, The effect of IGF‐II on the cell survival of AM5‐F and AM7‐F cells. The cells were treated with 100 ng/mL of IGF‐II for 72 h. MTT assay was performed as described above. Asterisk (*) indicates statistically significant difference at *P* < .05 (n = 3). C, Western blot analysis showing the effect of IGF‐II (1000 ng/mL) on the phosphorylation levels of p44/42 (Thr202/Tyr204), Akt (Thr308 and Ser473), and RSK1/p90 (Ser380). GAPDH was used as an internal control. Phospho‐p44/42 levels are expressed relative to the protein levels found in the untreated cells, which were arbitrarily defined as 1

Since microarray analysis detected the upregulation of various secreted proteins related to cell survival and proliferation (Tables S3 and S4), we examined the effect of recombinant proteins (Table S7) on the proliferation of AMU‐AM1 cells. Interestingly, MTT assay showed that IGF‐II significantly increased the proliferation of the patient‐derived AM5‐F and AM7‐F cells as well as AMU‐AM1 cells, compared to untreated cells (Figure 4A,B). Accordingly, IGF‐II increases the phosphorylation level of Erk1/2 (p44/42) and RSK1 (Figure 4C), suggesting that IGF‐II is a candidate cell growth factor for AMU‐AM1 cells.

### 3.5 Activation of TLR2 signaling in ameloblastoma cells

**Figure 5:**
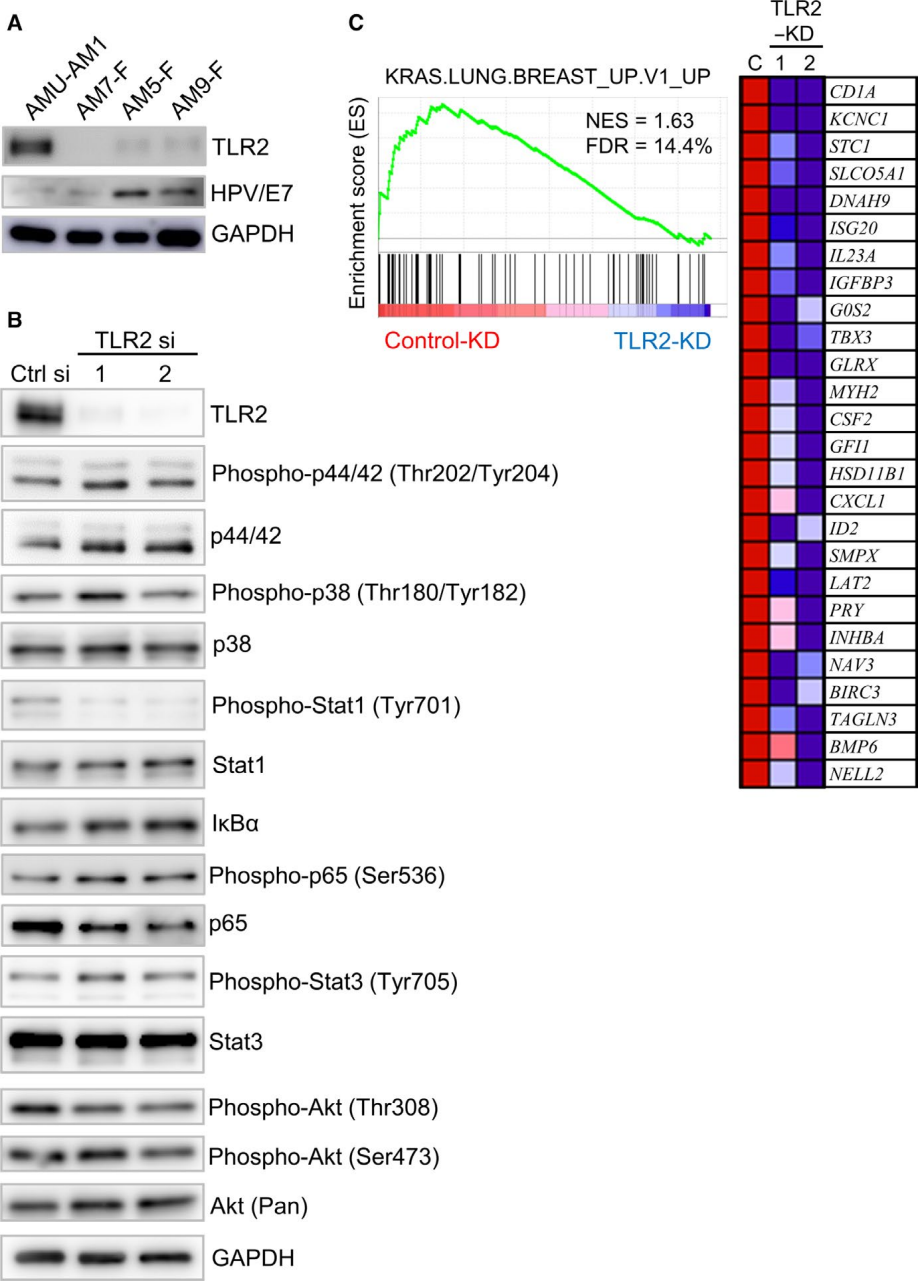
TLR2‐mediated signaling in AMU‐AM1 cells. A, Western blot analysis of the protein levels of TLR2 and HPV/E7 in AMU‐AM1 and ameloblastoma‐associated fibroblast (AAF) cells (AM5‐F, AM7‐F, and AM‐9F). B, Western blot analysis of the proteins under *TLR2* knockdown in the AMU‐AM1 cells. AMU‐AM1 cells were transfected with 20 nmol/L of control siRNA (Control si), *TLR2* siRNA‐1 (TLR2 si‐1), or *TLR2* siRNA‐2 (TLR si‐2) and then incubated for 48 h. The antibodies used for western blotting are shown in Table S2. GAPDH was used as an internal control. C, cDNA microarray analysis was performed using total RNA, which was isolated from AMU‐AM1 cells under control or *TLR2* knockdown. A representative GSEA enrichment plot from oncogenic signatures (C6) KRAS.LUNG.BREAST_UP.V1_UP with corresponding heat map images of the indicated gene sets is shown

We further investigated the highly upregulated (FC > 3.0) or downregulated genes (FC < 0.5) in the ameloblastoma group (ameloblastoma tumors and AMU‐AM1 cells) (Table S8). We found that TLR2, a gene involved in KRAS signaling, was listed as a top‐ranking upregulated gene in the ameloblastoma group (Figure 2A,B, Figure 5A, and Figure S5A). We examined the effect of *TLR2* knockdown on the cellular signaling pathway in AMU‐AM1 cells (Figure 5B and Figure S5B). Of note, GSEA of genes with oncogenic signatures clearly showed that *TLR2* knockdown significantly inactivates KRAS‐responsive genes compared to control cells (Figure 5C; eg *IGFBP3*, *INHBA*, and *CXCL1*; Table S9), suggesting that TLR2 expression play an important role in cell survival signaling in AMU‐AM1 cells. To further clarify the biological interaction of ameloblastoma cells with AAF, we examined the effect of various secreted proteins, which were predominantly expressed in ameloblastoma tumors and AMU‐AM1 cells, on the proliferation of AAFs. MTT assay showed that the cell survival of AAFs increases after treatment with IGF‐I, FGF‐2, and Midkine, and decreases after treatment with TGF family proteins, including growth differentiation factor 15 (GDF15), TGF‐β1, and TGF‐β3 (Figure S6A). In addition, we examined the effect of culture medium (CM), which was obtained from AMU‐AM1 cells in the presence or absence of Pam_3_CSK_4_, on the cell survival of AAFs. MTT assay showed that the percentage of cell survival of AAFs significantly increases in the presence of CM compared, regardless of Pam_3_CSK_4_ stimulation (Figure S6B). These results suggest that AMU‐AM1 cells may not only enhance the cell survival of themselves, but also may maintain that of associated fibroblasts by mediating secreted factors.

### 3.6 Role of TLR2 in the pathophysiological cell signaling of ameloblastoma cells

**Figure 6:**
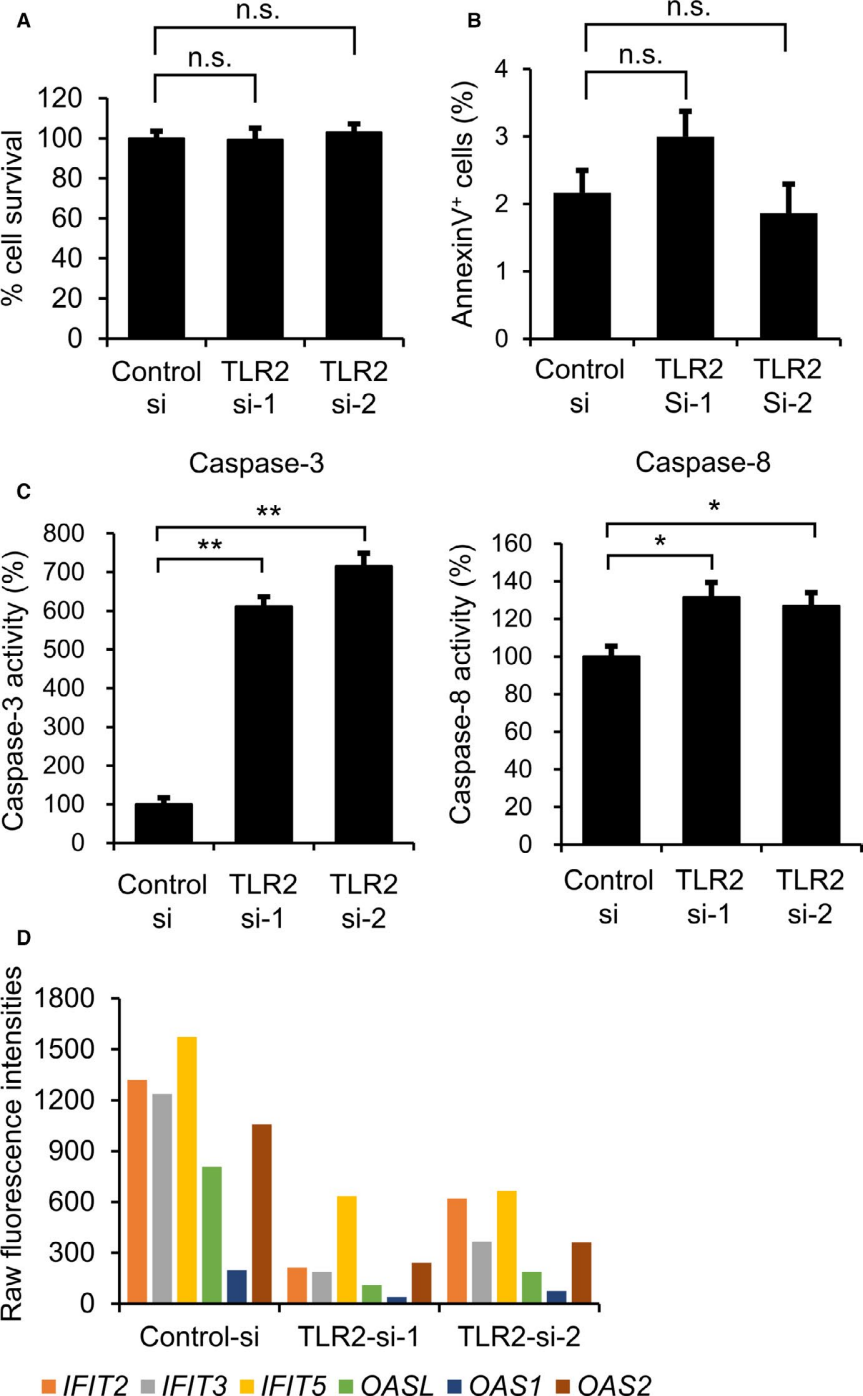
TLR2 expression is associated with caspase activities and IFN‐inducible gene expression. A‐C, Effect of TLR2 knockdown on cell survival (A), apoptosis (B), and caspase activities (C). A, The cell survivals are measured using the MTT assay. The cells were transfected with each siRNA and were further incubated for 72 h. Data are expressed relative to the mean optic density (595 nm) of cells under control siRNA treatment, which was arbitrarily defined as 100%. Data are expressed as mean ± SE (n = 3). B, Apoptotic cells were measured using AnnexinV‐FITC and a flow cytometry instrument. The bar graph shows the percentage of AnnexinV‐positive cells following 48 h of siRNA transfection. C, Caspase activities were measured using the Caspase‐Glo assay. The cells were transfected with each siRNA and were further incubated for 72 h. Data are expressed relative to the mean luminescence of cells under control siRNA treatment, which was arbitrarily defined as 100%. Data are expressed as mean ± SE (n = 3). Asterisks (* or **) indicate a statistically significant difference at *P* < .05 or *P* < .005, respectively (n = 3). D, The differential expression levels of interferon‐inducible genes (*IFIT2*, *IFIT3*, *IFIT5*, *OASL*, *OAS1*, and *OAS2*) are shown. Raw fluorescence values obtained by scanning were utilized for the comparison of gene expression between control knockdown and *TLR2* knockdown

We further examined the effect of TLR2 knockdown on the cell survival and cell death signaling. MTT and Annexin V assays showed that no significant differences in cell survival or in apoptosis were observed between cells with *TLR2* knockdown and control cells (Figure 6A,B). Intriguingly, *TLR2* knockdown significantly increased caspase‐3 and caspase‐8 activities but not caspase‐9 activity compared to control cells (Figure 6C), suggesting that TLR2‐mediated signaling may regulate caspase activity. In addition, *TLR2* knockdown readily inactivates inflammatory response‐related genes including C‐C motif chemokine ligand 2 (*CCL2*), CXC motif chemokine ligand 6 (*CXCL6*), and interleukin 6 (IL6) (Figure S7A), TLR signaling pathway‐related genes including interferon beta 1 (*IFNB1*), tumor necrosis factor (*TNF*), and TANK binding kinase 1 (*TBK1*) (Figure S7B), interferon response genes including 2'‐5'‐oligoadenylate synthetase like (*OASL*), interferon‐induced protein 44 (*IFI44*), and caspase 1 (*CASP1*) (Figure S7C), and TNF‐α signaling‐related genes including *CCL2*, *TNF*, and *CXCL11* (Figure S7D) (Tables S10 and S11). Furthermore, *TLR2* knockdown decreases the gene expression of interferon‐induced protein with tetratricopeptide repeats (*IFIT*) genes, including *IFIT2, IFIT3*, and *IFIT5*, as well as *OAS* genes, including *OASL*, *OAS1*, and *OAS2* (Figure 6D). These results suggest that TLR2 may mediates innate immune signaling in AMU‐AM1 cells.

### 3.7 TLR2 agonist activates NF‐kB signaling pathway in AMU‐AM1 cells

**Figure 7:**
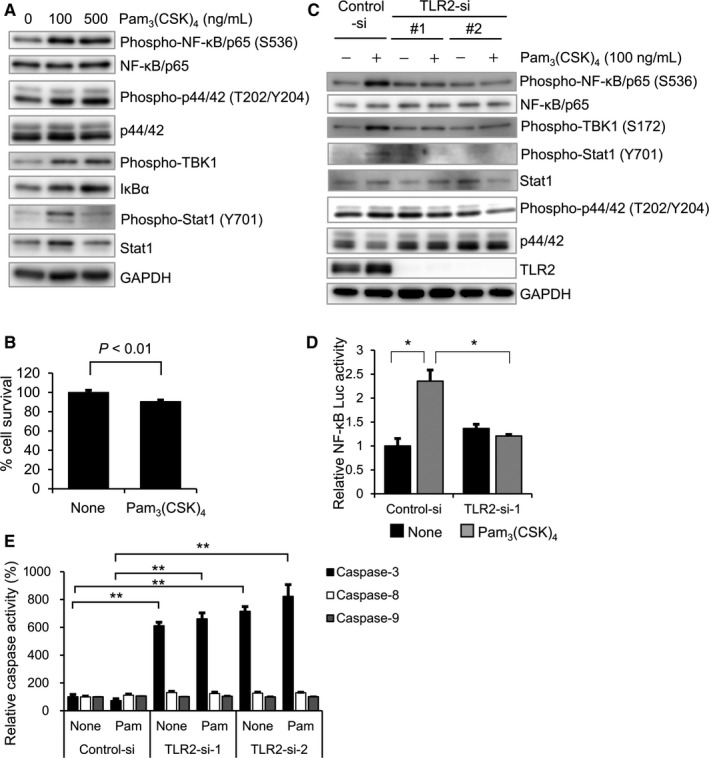
The effect of TLR2 agonist on TLR2 signaling in AMU‐AM1 cells. A, AMU‐AM1 cells were treated with a TLR2 agonist Pam_3_CSK_4_ (0, 100 and 500 ng/mL) for 2 h. Western blot analysis was performed as described in Figure 2B. B, AMU‐AM1 cells were incubated for 72 h in the presence or absence of Pam_3_CSK_4_ (100 ng/mL), and then subjected to MTT assay. Bar graph shows the relative mean optic density (595 nm) of untreated cells, which was arbitrarily defined as 100%. Data are expressed as mean ± SE (n = 3). C, The effect of TLR2 knockdown on the Pam_3_CSK_4_‐induced phosphorylation levels of p65, p44/42, TBK1, and STAT1. Each siRNA was introduced into AMU‐AM1 cells as described in Figure 5, and then incubated for 48 h. The cells were incubated for 2 h in the presence or absence of Pam_3_CSK_4_ (100 ng/mL), and then subjected to western blot analysis. D, The effect of TLR2 knockdown on the Pam_3_CSK_4_‐induced NF‐κB transcriptional activity using luciferase reporter assay. AMU‐AM1 cells were cotransfected with PBX‐II vector (containing NF‐κB binding sites; firefly luciferase) and the phRL‐TK vector (internal control; renilla luciferase) with control siRNA or TLR2 siRNA‐1 followed by treatment with Pam_3_CSK_4_ (100 ng/mL) for 8 h. After normalization to Renilla luciferase activity, the fold‐change relative NF‐κB transcriptional activity was expressed relative to that found in the untreated cells, which was arbitrarily defined as 1 (n = 4) (*P < .05). E, The effect of Pam_3_CSK_4_ on the activities of caspase‐3, caspase‐8, and caspase‐9 were measured using Caspase‐Glo assay. The cells were transfected with each siRNA and were further incubated for 48 h followed by treatment with Pam_3_CSK_4_ (100 ng/mL) for 24 h. Data are expressed relative to the mean luminescence of cells under control siRNA treatment, which was arbitrarily defined as 100%. Data are expressed as mean ± SE (n = 3). Asterisk (**) indicates statistically significant difference at *P* < .005

**Figure 8:**
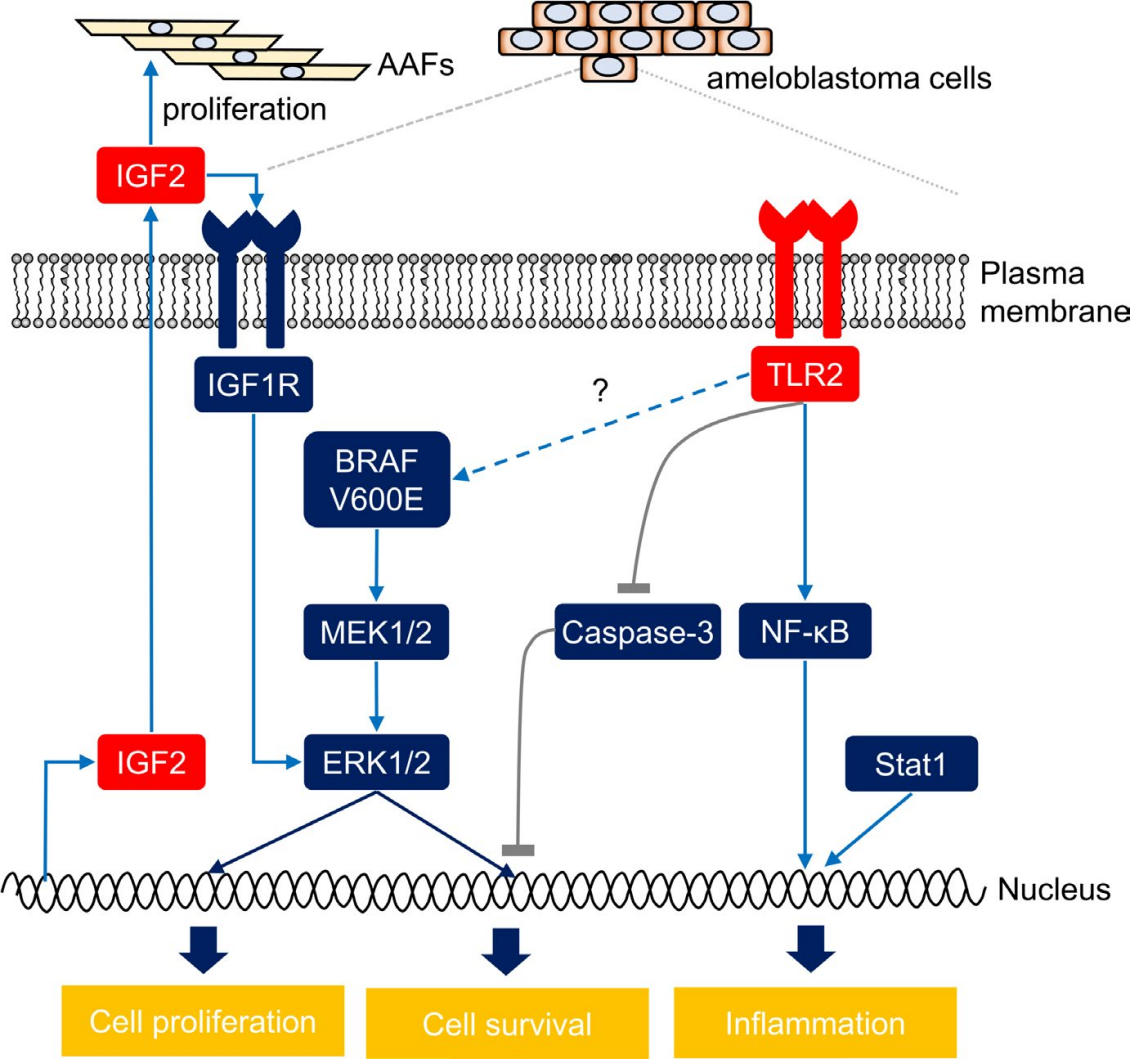
Schematic summary of cellular and molecular signaling pathways in ameloblastoma. In this study, our global transcriptome analysis identified significant upregulation of IGF2 and TLR2 in ameloblastoma tumors and AMU‐AM1 cells (AM cells). TLR2 knockdown suppressed the Pam_3_(CSK)_4_‐induced NF‐κB activity and inactivated KRAS‐responsive signaling, which was activated in most mandible ameloblastomas with *BRAF*/V600E gene mutations. TLR2 knockdown increased caspase activity regardless of ligand stimulation, suggesting that TLR2 may not only be involved in inflammation, but also in cell survival of ameloblastoma cells by mediating caspase activity. Furthermore, treatment with IGF2 increased the proliferation of both ameloblastoma‐associated fibroblasts (AAFs) and AMU‐AM1 cells, with concomitant augmentation of the phosphorylation level of Erk1/2, indicating that IGF2 may help the formation of ameloblastoma tumors

To clarify the role of TLR2 in ameloblastoma cells, we examined the effect of TLR2 agonist Pam_3_CSK_4_ on the cellular signaling in AMU‐AM1 cells. Treatment with Pam_3_CSK_4_ readily increases the phosphorylation levels of NF‐κB/p65, Erk1/2 (p44/42), TBK1, and Stat1 (Figure 7A). Surprisingly, Pam_3_CSK_4_ slightly but significantly decreases the cell survival of AMU‐AM1 cells (Figure 7B). *TLR2* knockdown decreases Pam_3_CSK_4_‐induced phosphorylation of p65 as well as TBK1 and Pam_3_CSK_4_‐induced NF‐κB transcriptional activity (Figure 7C,D). Pam_3_CSK_4_ has no effect on the activities of caspase‐3/7, caspase‐8, and caspase‐9, even with TLR2 knockdown (Figure 7E). Collectively, these results suggest that TLR2 signaling may mediate cell survival signaling in ameloblastoma cells (Figure 8).

## 4 DISCUSSION

Ameloblastoma is a benign tumor that sometimes exhibits malignant and/or cancerous properties. To delineate the molecular and cellular pathophysiology of ameloblastoma, we comprehensively investigated gene expression changes in patient‐derived ameloblastoma by comparing tumors with corresponding normal oral tissues. Our comprehensive cDNA microarray analysis revealed the upregulation of several growth factors and cytokines in ameloblastoma tumors and AMU‐AM1 cells. In addition, IGF‐II significantly increases the survival of both ameloblastoma cells and AAFs. We also found that TLR2 is highly expressed in ameloblastoma and may mediate both survival and inflammatory signaling in ameloblastoma cells. Furthermore, our data showed that midkine increases the proliferation of AAFs. These results suggest that ameloblastoma cells may control cell survival by mediating innate immune signaling.

In this study, we generated comprehensive gene expression data, which revealed the significant activation of oncogenic gene sets in the ameloblastoma tumor, such as KRAS‐responsive as well as EGFR‐induced genes. Recent NGS and immunohistochemistry analyses showing the high frequency of the *BRAF* V600E mutation in patients with ameloblastoma have implicated the involvement of the RAS‐MAPK pathway in the pathogenesis of ameloblastoma.^7^, ^8^ However, to date, global gene expression profiles in ameloblastoma remain poorly understood. In 2002, Heikinheimo et al investigated the expression of 588 cancer‐related genes in ameloblastoma patients.^22^ They showed increased expression of the activating protein‐1 (AP‐1) family of transcription factors (*FOS*, c‐fos proto‐oncogene; *JUNB*, jun B proto‐oncogene) and extracellular matrix (ECM)‐related genes (*MMP12*, *MMP 13*, and *COL8A1*). In our study, we did not find upregulation of the AP‐1 family genes; however, we did note the upregulation of ECM‐related genes including hyaluronan and proteoglycan link protein 1 (*HAPLN1*), *COL10A1*, *COL5A1*, and *COL12A1*, collagen triple helix repeat containing 1 (*CTHRC1*), *MMP1*, *MMP9*, and fibronectin type III domain containing 1 (*FNDC1*), in addition to *MMP12*, *MMP13*, and *COL8A1* (Table S3). Since ECM‐related molecules are associated with tumorigenesis by modulating tumor microenvironments, the upregulation of ECM‐related genes may play an important role in the formation of ameloblastomas. Moreover, our qRT‐PCR analysis, as well as microarray data, showed that mRNA expression levels of *INHBA*, *IGF2*, and *TLR2*, all of which are classified into the KRAS‐responsive gene sets, significantly increase in ameloblastoma compared to normal counterpart tissues. In contrast, no significant activation of PI3K‐Akt signaling was observed in ameloblastoma. These results strongly support the previous studies suggesting that RAS‐MAPK signaling pathway may play an important role in the pathogenesis of ameloblastoma. Although our microarray analysis uncovered global transcriptome data for ameloblastoma tumors, our data did not clarify genomic alterations, including gene amplification, genomic loss, and somatic mutations, in ameloblastomas. Therefore, further studies using NGS and/or RNA‐sequencing data would be useful for further understanding the pathogenesis of ameloblastoma.

There is a growing body of evidence that TLRs play a pivotal role in tumor survival and growth.^29^ Tye et al reported that upregulation of TLR2 promotes gastric tumorigenesis independent of tumor inflammation.^30^ Kim et al also reported that carcinoma‐produced factors activate myeloid cells through TLR2 to stimulate metastasis.^23^, ^31^ In this study, we showed that (a) TLR2 knockdown decreases gene expression related to KRAS‐responsive gene set and inflammatory gene sets, (b) TLR2 knockdown decreases NF‐κB signal transduction after ligand stimulation, and (c) TLR2 knockdown increases caspase activities. Although our data does not always show the role of TLR2 in cell growth of ameloblastoma, TLR2 may proactively function in ameloblastoma cells. These results suggest that TLR2 maintains the survival of ameloblastoma cells in cooperation with other TLR molecules, which might activate intracellular signaling by mediating common adaptor molecules, including MyD88. Since AMU‐AM1 cells possess the *BRAF* V600E mutation, it is also possible that proactive RAS‐MAPK signaling cooperates with TLR2 to maintain cell survival.

TLR2 mediates the innate immune response by recognizing both exogenous and endogenous ligands, and plays an important role in innate immunity.^32^, ^33^, ^34^, ^35^ We found that stimulation of TLR2 with Pam_3_CSK_4_ increased phosphorylation levels of NF‐κB and its transcriptional activity; these increases were completely abrogated by TLR2 knockdown. These results suggest that engagement of exogenous ligands to TLR2 may enhance inflammatory and/or cell survival signaling in ameloblastoma cells. Interestingly, treatment with Pam_3_CSK_4_ decreases the cell survival of AMU‐AM1 cells, while the TLR1/2 antagonist CPT‐Cu‐22 increases cell survival. Indeed, stimulation and/or inhibition of TLR2 would be challenging as a novel therapeutic approach for cancer therapy.^36^, ^37^, ^38^ Hence, these results suggest that modulation of TLR2 signaling may affect the tumor environment and survival of ameloblastoma cells.

It has been reported that FGF‐7 and FGF‐10 are involved in ameloblastoma proliferation via the MAPK pathway.^19^ In this study, our comprehensive gene expression analysis showed that a number of growth factors, cytokines, and chemokines were significantly increased in ameloblastoma compared to corresponding normal tissues. Indeed, we found that the expression level of IGF‐II significantly increases in AMU‐AM1 cells, and that stimulation with IGF‐II augments the proliferation of AMU‐AM1 cells. IGF‐II can promote many types of normal as well as cancer cells. Although IGF2 is not likely a specific growth factor for ameloblastoma, our data are the first to demonstrate the positive effect of IGF2, which is classified into KRAS‐responsive gene sets, on ameloblastoma cells. These results suggest that RAS‐RAF‐MAPK pathway promotes cell growth of ameloblastoma by mediating IGF2 stimulation. In addition, treatment with midkine increases the cell survival of AAFs. AMU‐AM1‐derived CM increases the survival of AAFs. This result further supports the hypothesis that ameloblastoma cells positively regulate the cell survival of tumor‐associated fibroblasts by secreting growth factors. Hence, these results indicate the possibility that ameloblastoma‐derived secreting factors may closely associated with the tumor cell growth and/or the tumor environment of ameloblastoma.

On the contrary, amelogenesis is physiologically important to maintain oral health and prevent diseases.^39^, ^40^ Several molecules have been reported to play an essential role in amelogenesis. *AMTN* is normally expressed and secreted predominantly by ameloblasts during the transition and maturation stages.^41^ Lacruz et al reported that overexpression of *AMTN* under the amelogenin promoter causes irregular enamel surface structure and disrupts the normal Tomes’ process in mice.^42^ In this study, our gene expression analysis revealed overexpression of *AMTN* in ameloblastoma and AMU‐AM1 cells, suggesting two possibilities: (a) the origin of ameloblastoma might be ameloblasts in the maturation stage; and (b) ectopic secretion of *AMTN* from ameloblastoma might lead to disorganized enamel structure and development of an ameloblastoma tumor. Although the relationship between *AMTN* overexpression and development of ameloblastoma is still unknown, our finding might provide novel insight into the molecular pathogenesis of ameloblastoma for future research.

TGF‐β signaling is thought to mediate the differentiation of epithelial cells into functional ameloblasts.^41^, ^42^ It has also been reported that TGF‐β signaling is related to tumor progression.^43^, ^44^ In this study, we found that genes related to the TGF‐β signaling pathway are activated in ameloblastoma. We also found that expression levels of TGF‐β family genes, including *TGFB1*, *TGFB3*, and *GDF15*, significantly increased in ameloblastoma. Stimulation with the recombinant proteins did not change the survival of AMU‐AM1 cells but significantly decreased the survival of AAFs. Although expression of TGF‐β family proteins has been observed in normal ameloblast cells, TGF‐β signaling may be associated with the tumorigenesis of ameloblastoma. Since some patients with ameloblastoma are reported to experience relapses and/or invasive phenotypes, it would be of particular interest to investigate whether upregulated TGF‐β‐related proteins are involved in the migration/invasion properties of ameloblastoma cells.

In conclusion, this study is the first to show global transcriptomic data in ameloblastoma tumors. Our gene expression analyses clearly indicated the overexpression of TLR2 with a concomitant activation of the KRAS‐related oncogenic gene set in patients with ameloblastoma. Although use of only single cell line AMU‐AM1, our finding that TLR2 knockdown suppresses KRAS‐related signaling and immune responses in AMU‐AM1 cells strongly indicates the possibility that TLR2 plays a pivotal role in ameloblastoma. Since TLR2 is highly expressed in ameloblastoma tumors and AMU‐AM1 cells, which are derived from patients with recurrent ameloblastoma, it would be of particular interest to examine the association of the level of TLR2 expression with ameloblastoma malignancy. Our data indicate the possibility that TLR2 would be a candidate molecule for developing a treatment for ameloblastoma. Additional studies are needed to better understand the involvement of the TLR signaling pathway in ameloblastoma tumor development. Our findings may help to further clarify the pathophysiology of ameloblastoma and lead to the development of precision medicine for patients with ameloblastoma.

## CONFLICT OF INTEREST

The authors declare no competing interests.

## AUTHOR CONTRIBUTIONS

AO, TO, YH, and YK conceived, proposed, and designed the study. S.Karnan., WM, TH, M.L‐R., HK, and ST developed the methodology. S.Kondo, AO, S.Karnan., and WM acquired the experimental data. WM, TH, and M.L‐R. provide the technical supports. S.Kondo, AO, TO, SK, WM, TH, and M.L‐R. analyzed and interpreted the data. S.Kondo, TO, KI, AF, TH, and YK obtained the informed consent from each patient, performed the sample collection, and provided material support. S.Kondo, AO, and YH wrote manuscript. AO, YH, and YK supervised the study.

## Data Availability

The data that support the findings of this study are available in the Gene Expression Omnibus (GEO) at National Center for Biotechnology Information [https://www.ncbi.nlm.nih.gov/geo/], reference number GSE132472. These data were derived from the following resources available in the public domain: https://www.ncbi.nlm.nih.gov/geo/query/acc.cgi?acc=GSE132472
