# Supplementary material for: Discovery of novel molecular characteristics and cellular biological properties in ameloblastoma
Source: Cancer Med. 2020 Feb 25;9(8):2904–17. doi: 10.1002/cam4.2931 (PMC7163100; doi:10.1002/cam4.2931)
Supplement: Supplementary file 1 [file CAM4-9-2904-s001.pdf]

BRAF exon 15

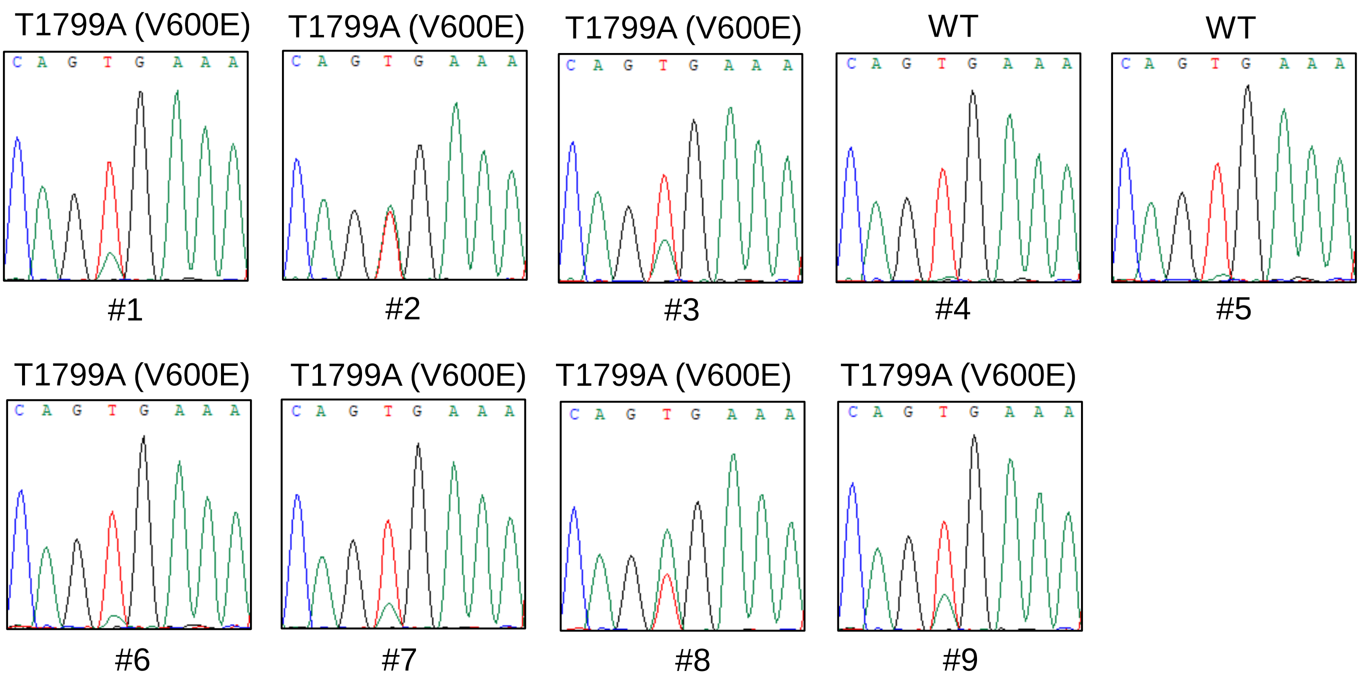

Supplementary Fig. S1. **Sanger DNA sequencing analysis of genomic DNA from the ameloblastoma patients**. The isolated DNA were amplified using a *BRAF* (Exon 15) specific gene primer set (listed in Supplementary Table S1).

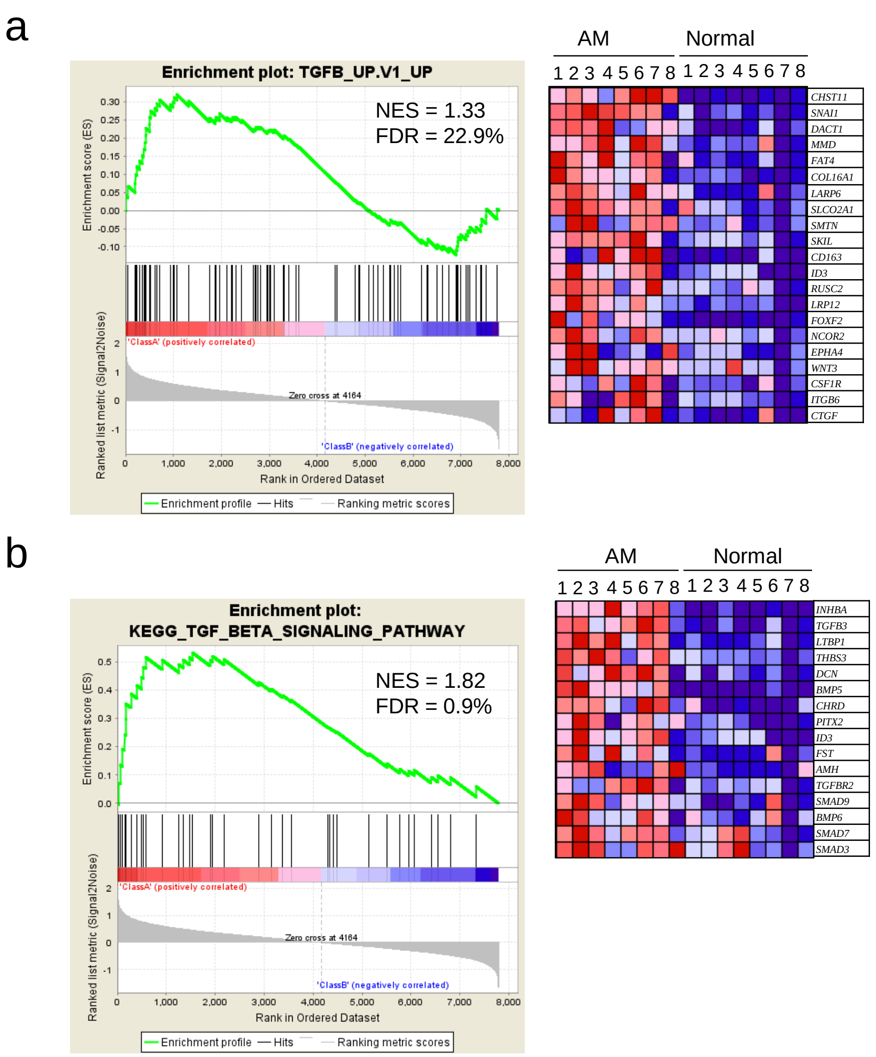

Supplementary Fig. S2. **Activation of genes related to the TGF-beta signaling pathway gene sets in ameloblastoma tumor**. (A-B) cDNA microarray analysis were performed using total RNA, which were isolated from tumor lesion or normal oral tissues of 8 individual patients with ameloblastoma. Gene set enrichment analysis (GSEA) was conducted using GSEA v2.2.4 software and Molecular Signatures Database (Broad Institute). All raw data were formatted and applied to oncogenic signatures (c6.all, **a**) and KEGG genes (C2.cp.kegg, **b**). Representative enrich with corresponding heatmap images of the indicated gene sets in the ameloblastoma tumor (AM) and/or normal tissues (Normal) are shown. Genes contributing to enrichment are shown in rows. Expression level is represented as a gradient from high (red) to low (blue). FDR, false discovery rate; NES, normalized enrichment score.

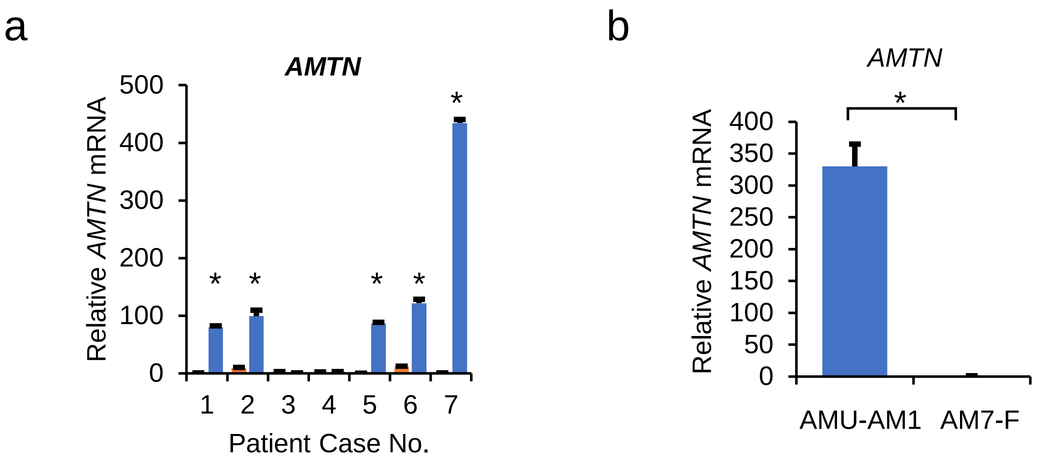

Supplementary Fig. S3. Expression of *AMTN* (amelotin) gene in ameloblastoma tumor and AMU-AM1 cells.
a. qRT-PCR analysis for the expression level of *AMTN* in ameloblastoma and normal oral tissues. **b**. The expression level of *AMTN* in AMU-AM1 cells and the patient-matched fibroblast cell line AM-7F cells. The primers used for qRT-PCR are shown in Table S1. Relative gene expression levels are shown after normalization to *GAPDH* mRNA expression. The data are expressed relative to the mRNA levels found in the corresponding normal tissue sample of patient No.1, which was arbitrarily defined as 1. The values shown represent the mean ± SE (n=3).

Supplementary Fig. S4

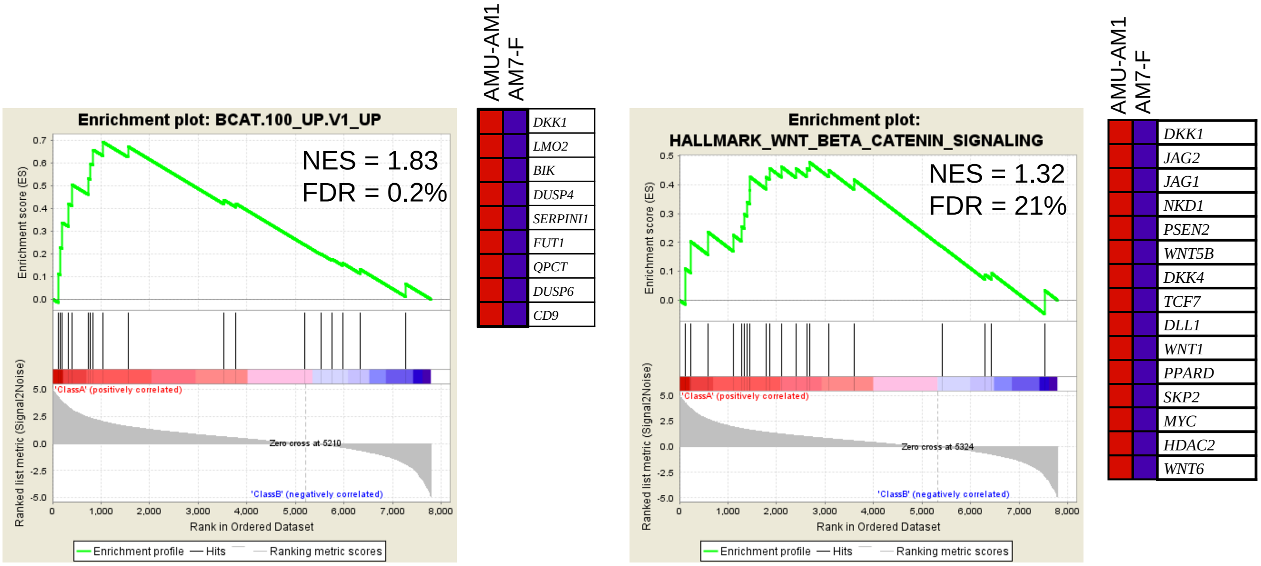

Supplementary Fig. S4. **Activation of genes related to the β-catenin signaling pathway gene sets in AMU-AM1 cells**. (A-B) cDNA microarray analysis were performed using total RNA, which were isolated from cultivated AMU-AM1 cells and AM-7F cells. Gene set enrichment analysis (GSEA) was conducted using GSEA v2.2.4 software and Molecular Signatures Database (Broad Institute). All raw data were formatted and applied to oncogenic signatures (c6.all, **a**) and HALLMARK genes (h.all, **b**). Representative enrich with corresponding heatmap images of the indicated gene sets in the AMU-AM1 cells and AM-7F cells are shown. Genes contributing to enrichment are shown in rows. Expression level is represented as a gradient from high (red) to low (blue). FDR, false discovery rate; NES, normalized enrichment score.

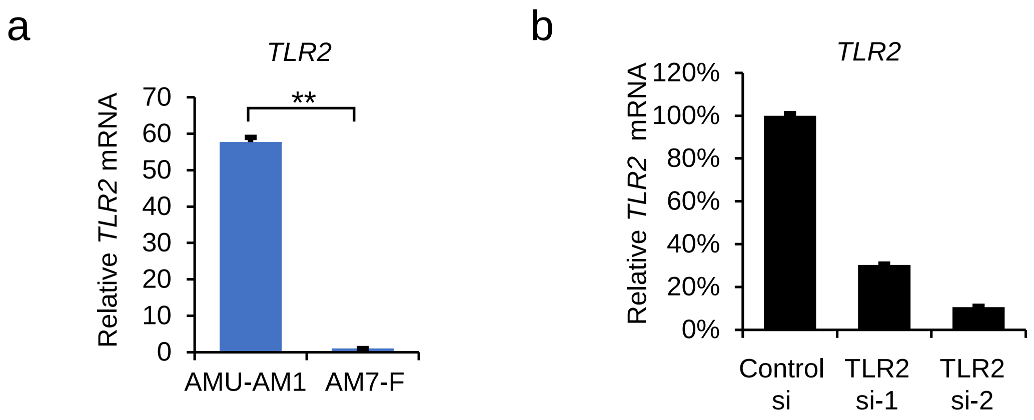

Supplementary Fig. S5. **Expression of TLR2 in AMU-AM1 cells and ameloblastoma-associated fibroblast (AAF) cells**. **a**, qRT-PCR analysis of the mRNA level of in AMU-AM1 and ameloblastoma-associated fibroblast (AAF) cells (AM5-F, AM7-F and AM-9F cells). The data are expressed relative to the mRNA levels found in the AM7-F cells, which was arbitrarily defined as 1 (n = 3). **b**, qRT-PCR analysis of mRNA level of *TLR2* in the AMU-AM1 under TLR2 knockdown. AMU-AM1 cells were transfected with 20 nM of control siRNA (Control si), TLR2 siRNA-1 (TLR2 si-1) or TLR2 siRNA-2 (TLR si-2), and then incubated for 48 h. The data are expressed relative to the mRNA levels found in the cells under knockdown with control siRNA, which was arbitrarily defined as 1 (n = 3).

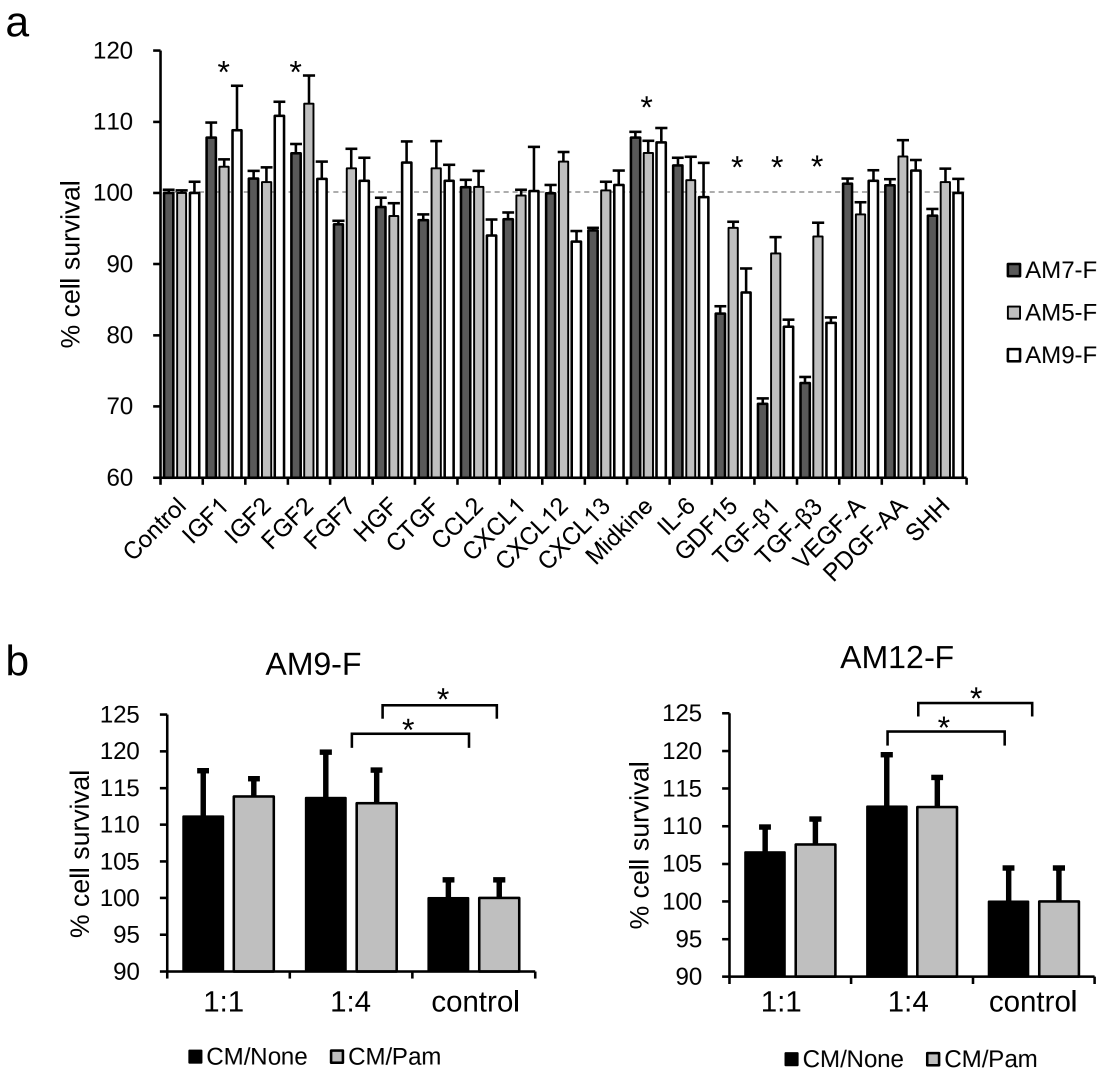

Supplementary Fig. S6. Effect of growth factors on the proliferation of ameloblastoma-associated fibroblasts (AAFs). **a**, MTT assay showing the effect of various growth factors on the cell survival of AAFs (AM-5F, AM-7F and AM-9F). AAFs (2×10^3 cells/well in a 96-well plate) were treated as described in Fig. 4A. **b**, MTT assay showing the effect of cell culture medium of AMU-AM1 on the survival of AAFs in the presence or absence of $Pam_3(CSK)_4$ (100 ng/mL). The cell culture medium was diluted at 1:1 or 1:4 with fresh culture medium. The AAFs cells Data are expressed relative to the mean optic density (595 nm) of untreated cells, which was arbitrarily defined as 100%. Data are expressed as mean ± SE (n = 3). Asterisk (*) indicates statistically significant difference at $P < 0.05$ (n = 3).

Supplementary Fig. S7

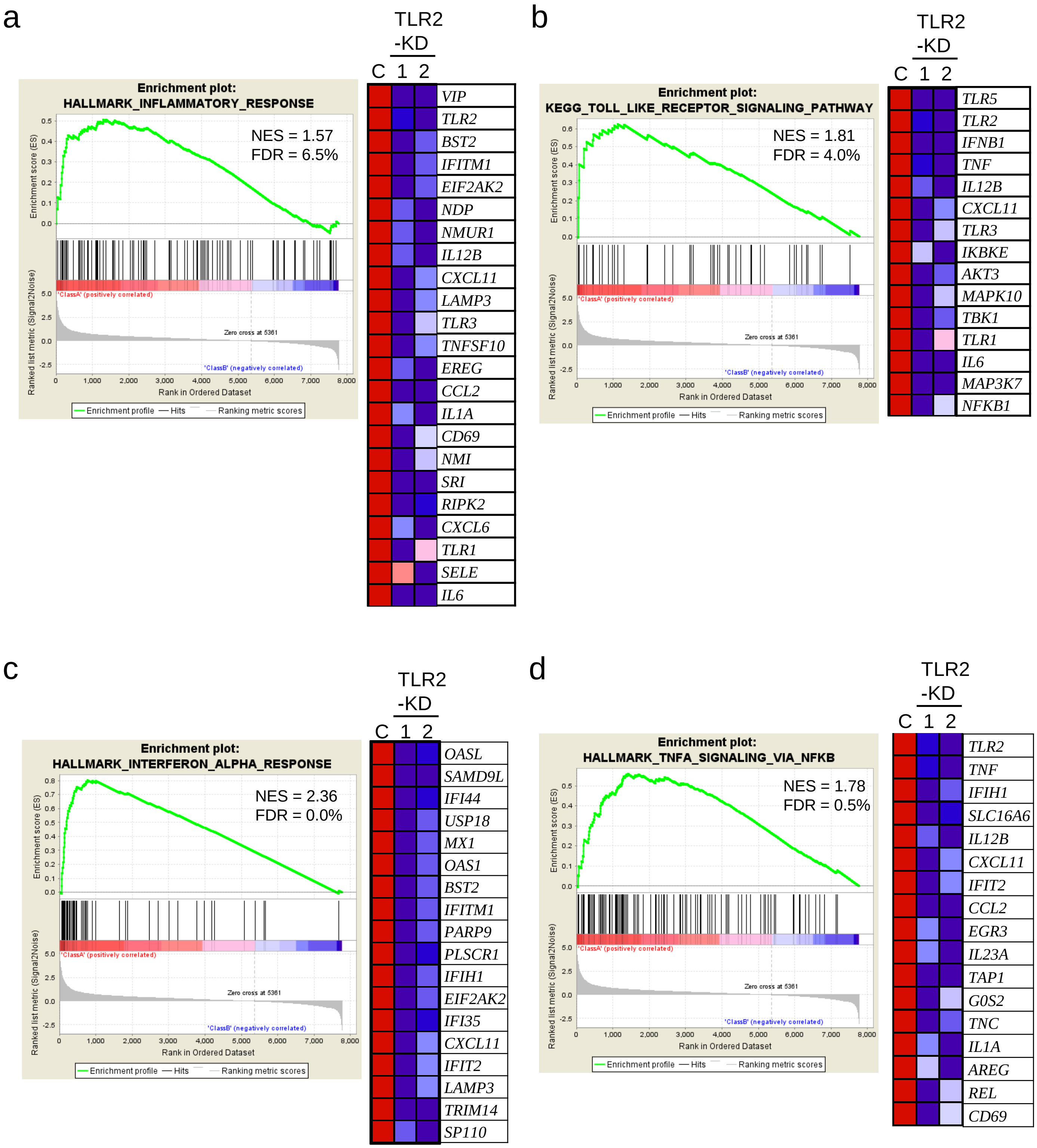

Supplementary Fig. S7. Effect of *TLR2* knockdown on the inflammatory response and innate immunity in AMU-AM1 cells. (**a-d**) cDNA microarray analysis were performed using total RNA, which were isolated from cultivated AMU-AM1 cells under control or *TLR2* knockdown. Gene set enrichment analysis (GSEA) was conducted using GSEA v2.2.4 software and Molecular Signatures Database (Broad Institute). All raw data were formatted and applied to HALLMARK genes (h.all, **a, c-d**) and KEGG genes (C2.cp.kegg, **b**). Representative enrich with corresponding heatmap images of the indicated gene sets in the AMU-AM1 cells are shown. Genes contributing to enrichment are shown in rows. Expression level is represented as a gradient from high (red) to low (blue). FDR, false discovery rate; NES, normalized enrichment score.
